# A Genome-Wide Association Study on Obesity and Obesity-Related Traits

**DOI:** 10.1371/journal.pone.0018939

**Published:** 2011-04-28

**Authors:** Kai Wang, Wei-Dong Li, Clarence K. Zhang, Zuoheng Wang, Joseph T. Glessner, Struan F. A. Grant, Hongyu Zhao, Hakon Hakonarson, R. Arlen Price

**Affiliations:** 1 Center for Applied Genomics, Children's Hospital of Philadelphia, Philadelphia, Pennsylvania, United States of America; 2 Zilkha Neurogenetic Institute, Department of Psychiatry and Department of Preventive Medicine, University of Southern California, Los Angeles, California, United States of America; 3 Center for Neurobiology and Behavior, Department of Psychiatry, University of Pennsylvania, Philadelphia, Pennsylvania, United States of America; 4 WM Keck Laboratory, Biostatistics Division, Yale University School of Medicine, New Haven, Connecticut, United States of America; 5 Department of Biostatistics, Yale University, New Haven, Connecticut, United States of America; 6 Department of Pediatrics, University of Pennsylvania, Philadelphia, Pennsylvania, United States of America; Vanderbilt University Medical Center, United States of America

## Abstract

Large-scale genome-wide association studies (GWAS) have identified many loci associated with body mass index (BMI), but few studies focused on obesity as a binary trait. Here we report the results of a GWAS and candidate SNP genotyping study of obesity, including extremely obese cases and never overweight controls as well as families segregating extreme obesity and thinness. We first performed a GWAS on 520 cases (BMI>35 kg/m^2^) and 540 control subjects (BMI<25 kg/m^2^), on measures of obesity and obesity-related traits. We subsequently followed up obesity-associated signals by genotyping the top ∼500 SNPs from GWAS in the combined sample of cases, controls and family members totaling 2,256 individuals. For the binary trait of obesity, we found 16 genome-wide significant signals within the *FTO* gene (strongest signal at rs17817449, P = 2.5×10^−12^). We next examined obesity-related quantitative traits (such as total body weight, waist circumference and waist to hip ratio), and detected genome-wide significant signals between waist to hip ratio and *NRXN3* (rs11624704, P = 2.67×10^−9^), previously associated with body weight and fat distribution. Our study demonstrated how a relatively small sample ascertained through extreme phenotypes can detect genuine associations in a GWAS.

## Introduction

Obesity is the sixth most important risk factor contributing to the overall burden of disease worldwide [1]. Affected subjects have reduced life expectancy, and they suffer from several adverse consequences such as cardiovascular disease, type 2 diabetes and several cancers. Many studies have shown that body weight and obesity are strongly influenced by genetic factors, with heritability estimates in the range of 65–80% [2], [3]. Genetic variants in several genes are known to influence BMI, but these mutations are rare and often cause severe monogenic syndromes with obesity [4]. With the development of high-throughput genotyping techniques and the implementation of genome-wide association studies (GWAS), common variations, such as those in *FTO* [5] and *MC4R* [6], have been associated with obesity and body mass index (BMI). Recent large-scale meta-analysis of multiple GWAS identified additional genes harboring common SNPs that associate with BMI [7]–[10]. GWASs have also found associations with measures of body fat distribution [9], [11], [12]. By far the largest GWAS to date included almost 250 thousand individuals and 2.8 million SNPs [13]. Associations of BMI with 28 loci reached genome wide significance, including 10 that were reported previously and 18 that were newly identified. Four additional loci were associated with body fat distribution, all of which had been identified previously. However, even this major expansion of sample size has not explained much variation, 1.39% for BMI and 0.16% for body fat distribution. On the other hand, confirmation of existing BMI loci, and detailed analysis on their association with obesity as a binary trait and with other obesity-related quantitative traits, are important at the current stage to move GWAS signals forward and understand their functional consequences.

A few studies utilized samples with early-onset or morbid obesity for discovery, and replicated previously reported association signals on BMI [7], [14]–[16], or implicated specific genetic variants such as a recurrent 16p11.2 deletion [17]. Utilizing extreme phenotypes increases the odds ratio of association, with improved power to identify novel association signals under fixed genotyping budgets and fixed sample sizes. We have collected a large cohort of obese cases and families ascertained from tails of BMI distribution together with detailed phenotype measures on multiple obesity-related traits. In addition, we have adult controls who have never been overweight. However, given fixed genotyping budget, instead of genotyping all these samples by whole-genome SNP arrays, we elected to perform a case-control GWAS, and then follow up the top signals by candidate SNP genotyping on the entire set of samples including family members. Therefore, the unique dataset provides an opportunity to examine GWAS associations in a combined sample of cases, family members, and controls.

## Results

**Figure 1:**
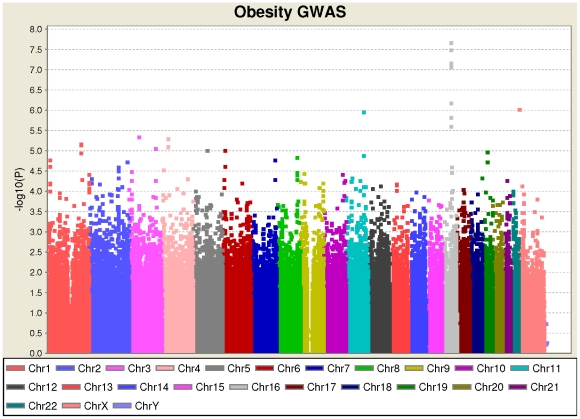
Manhattan plot (logarithm of P-values versus chromosome coordinates) for SNP association on obesity. The *FTO* locus on 16q12.2 reached genome-wide significance.

We analyzed genotype data for 520 cases and 540 control subjects, and performed a GWAS on obesity as a binary trait. We observed a strong association of obesity to the *FTO* gene, with the most significantly associated marker being rs3751812 (P = 2.01×10^−8^, odds ratio = 1.64). All association signals with P<10^−5^ are shown in **Table 3**, and the Manhattan plot is shown in **Figure 1**. No additional loci with genome-wide significance were identified in the GWAS; nevertheless, the fact that *FTO* readily reached genome-wide significance in a small data set confirmed the high quality of the phenotypes within the sample collection. It also illustrated how a small sample ascertained from extreme phenotypes have high power to detect genuinely associated genes, compared to quantitative trait association analysis conducted on population-based samples.

Considering the possibility that some obesity-associated genes may be enriched among the top ranked genes in GWAS, we next followed up selected association signals (∼500 top SNPs) by iSelect genotyping on 2,256 cases, family members and controls. Additionally, we also performed dense genotyping of 49 SNPs in the *FTO* gene itself, including 7 from the original top 500 and 42 spanning the entire gene. We used the MQLS software for the association analysis, to account for the familial relationships. Interestingly, the significance for *FTO* SNPs increased by several orders of magnitude (P-values range from 10^−9^ to 10^−12^), suggesting that genotyping additional family members increased power to detect genuine associations (**Table 4**).

We next completed exploratory analyses of quantitative measures of obesity. In part because of the extreme bimodality of the phenotype distributions based on sample ascertainment, we controlled for case/control status. Moreover, this approach makes it possible to assess the potential effects of genes on the extent of obesity in extremely obese individuals. The complete set of results (P<1×10^−6^) were given in **Table 5**. We note that a few markers reached P<5×10^−8^, notably waist circumference (chromosome 21, rs11088859, p = 3.75×10^−8^, nearest gene *NCAM2*), and waist to hip ratio (chromosome 14, rs11624704, p = 2.67×10^−9^, nearest gene *NRXN3*), but the *NCAM2* locus cannot be regarded as genome-wide significant considering the need to adjust for multiple phenotypes being tested. Therefore, these exploratory results were provided as potentially interesting findings worthy of additional replication efforts.

**Table S1** summarizes our results with respect to previously reported associations with obesity related traits. As noted, only *FTO* and *NRXN3* reached genome wide significance. Three genes, including *SH2B1*, *MC4R* and *KCTD15*, showed trends towards significance in the case/control association tests (P-value ranges from 0.015 to 0.065), but they did not pass multiple testing thresholds (based on number of genes tested). For quantitative traits, we cannot estimate the power of our study, since previously published studies utilized population samples for quantitative trait association with BMI [18]. Nevertheless, in our data, it is interesting to see that *MC4R* and *FTO* are the two genes with the strongest effect sizes (risk allele odds ratio >1.2), probably explaining why they were the first two genes identified in GWAS for BMI [5], [6].

## Discussion

In the current study, we performed a GWAS on obesity and obesity-related traits. The *FTO* gene reached genome-wide significance in this cohort with an odds ratio of 1.6. The *MC4R* gene is the second gene found by GWAS to be associated with BMI [6], and, while only marginally associated with obesity in our study, its odds ratio was 1.3. Given our modest sample size in GWAS, we estimate that the power to detect association (with perfect SNP tagging) at P<5×10^−8^ is 78.8% and 0.18% for *FTO* and *MC4R*, respectively. These odds ratio estimates in our data are higher than previous reports, for example, odds ratio for *FTO* is 1.3 in a study for early-onset obesity [5], for *FTO* and *MC4R* are 1.46 and 1.02 in a study for extreme obesity [14], or 1.25 and 1.26 in a study on morbidly obese adults with familial obesity [15], or 1.27 and 1.12 for obesity [10]. We note that the Hinney et al report investigated early onset extreme obesity and reported an odds ratio of 1.67 for *FTO* [16], comparable to our study. Therefore, the increased effect size could be due to the specific sample ascertainment scheme that we have used, that is, we sampled from the extreme tails of a quantitative trait distribution based on BMI. Even with the augmented sample of cases, family members and controls, no other SNPs reached genome wide significance. The results therefore strongly suggest that *FTO* and *MC4R* might be the only two major-effect genes for obesity with common variants in populations of European ancestry. Our study also represents an example where enrichment of extreme cases and controls can lead to increased odds ratio, and subsequently leads to improved power to detect associations.

The association between waist to hip ratio and the *NRXN3* gene is of interest, as this is the third time the gene has been associated with body fat distribution [12], [13]. Neurexins are expressed in nervous tissue and are thought to be involved in cell adhesion during synapse formation [19]. Besides fat distribution, *NRXN3* has been associated with several other traits, including addictions and schizophrenia [20]–[22]. Identifying the specific causal variant may be difficult because *NRXN3* is an extremely large gene (∼1.5 Mb) [19]. It is controlled by two promoters and has multiple transcripts. The SNPs associated with weight and fat distribution lie in different parts of the gene and will likely involve different transcripts with potentially different functions. The associated SNP in our study, rs11624704, appears to be about 85 kb upstream of the first exon, while those for the previous two studies, rs10150332 and rs10146997, appear to be about 8 kb apart near exon 11.

In conclusion, we have assayed a sample collection of obese cases, families and never-overweight controls, and performed association analysis on obesity and multiple quantitative phenotype measures. We obtained strong support for *FTO* as well as suggestive confirmation of several previously identified BMI-associated genes in obesity. Another outcome of our study is the identification of new candidate genes for obesity-related traits. Of particular interest is the association of *NRXN3* with body fat distribution among extremely obese individuals.

## Materials and Methods

### Study participants

The current GWAS study includes 520 cases and 540 control subjects, who were non-Hispanic Caucasians. Cases were obese (BMI≥35 kg/m^2^) with a lifetime BMI>40 kg/m^2^. Among them, 32 were male while the rest were female subjects. Independent controls were selected who had a current and lifetime BMI≤25 kg/m^2^. The individuals in the samples were of approximately the same age but differed in average BMI by 29 kg/m^2^ (**Table 1**). After performing the GWAS, a combined sample of cases, controls and family members (N = 2,256), including all the study participants in the GWAS, were included for genotyping the top ∼500 most significant SNPs based on genotyping budget. Subject characteristics of family members were shown in **Table 2**. Note that this is a study originally designed for investigating obesity genes in female subjects, but over time we have included a small fraction of males during the recruitment. All subjects gave written informed consent, and the protocol was approved by the Committee on Studies Involving Human Beings at the University of Pennsylvania.

**Table 1:** Sample characteristics of 1,060 cases and controls in GWAS.

cases	N	Minimum	Maximum	Mean	SD
488 females	32 males				
BMI^1^	520	35.57	96.95	49.39	8.78
%FAT	467	30.70	70.70	49.88	6.03
WEIGHT	512	77.05	273.64	138.13	26.91
HEIGHT	509	135.60	198.30	167.07	7.87
WAIST	509	74.40	224.79	122.11	15.32
HIP	509	67.00	276.00	148.49	18.16
WHR	508	0.61	1.46	0.83	0.09
AGE	520	18	64	41.06	9.35
AGEONSET^2^	411	.5	55.0	13.63	9.02
**controls**					
532 females	8 males				
BMI*	540	15.97	24.93	20.75	1.76
%FAT	530	6.90	40.00	23.70	5.45
WEIGHT	539	37.50	90.00	55.52	6.39
HEIGHT	539	133.90	194.00	163.49	6.53
WAIST	539	40.20	100.00	72.89	5.76
HIP	539	56.90	110.10	86.51	6.51
WHR	538	0.50	1.33	0.84	0.07
AGE	540	16	65	43.10	8.81

1 : Self reported maximum BMI were used in 11 cases and 1 control.

2 : AGEONSET refers to the age of onset for the obesity diagnosis.

**Table 2:** Sample characteristics of 2,256 samples in the candidate SNP genotyping.

	N	Minimum	Maximum	Mean	SD
**Cases**					
	**546 females, 37 males**				
BMI	583	35.08	96.95	49.41	8.81
% FAT	529	30.70	70.70	50.01	5.92
WEIGHT	574	77.05	273.64	138.19	26.62
HEIGHT	571	135.60	198.30	167.10	7.83
WAIST	571	74.40	224.79	122.46	15.17
HIP	570	67.00	276.00	148.59	17.80
WHR	569	.61	1.46	.83	.09
AGE	583	18	64	41.06	9.42
AGEONSET^2^	460	1	55	13.96	8.98
**Controls**					
	**537 females, 8 males**				
BMI	545	15.99	24.93	20.77	1.79
%FAT	535	7.50	40.00	23.66	5.61
WEIGHT	544	37.50	90.00	55.55	6.66
HEIGHT	544	133.90	194.00	163.43	6.82
WAIST	544	40.20	100.00	72.78	5.74
HIP	544	56.90	110.30	86.84	6.94
WHR	543	.50	1.33	.84	.07
AGE	545	16	65	42.64	8.75
**Fathers** 1					
BMI	400	14.68	71.89	28.66	7.27
%FAT	326	3.30	59.20	25.97	8.53
WEIGHT	378	45.46	252.27	89.77	23.93
HEIGHT	375	153.70	198.12	176.89	7.12
WAIST	359	79.00	169.00	102.14	12.86
HIP	357	58.00	167.40	106.00	14.59
WHR	356	.72	1.66	.97	.07
AGE	400	42	95	66.74	9.85
AGEONSET^2^	152	1.0	74.0	40.93	18.37
**Mothers** 1					
BMI	411	15.94	69.02	32.77	10.37
%FAT	338	14.70	63.30	40.69	9.39
WEIGHT	392	40.45	185.23	87.76	28.53
HEIGHT	389	142.50	181.60	163.55	6.62
WAIST	380	61.80	159.00	96.97	19.91
HIP	379	60.40	174.90	116.21	21.42
WHR	379	.67	1.54	.83	.08
AGE	411	41	90	63.47	9.77
AGEONSET^2^	247	.5	79.0	30.23	19.60
**Sisters**					
BMI	278	17.01	77.58	34.34	11.23
%FAT	233	15.60	59.90	39.94	11.10
WEIGHT	268	45.68	212.95	94.74	31.57
HEIGHT	266	135.60	185.40	166.02	6.93
WAIST	260	60.90	165.80	97.48	21.45
HIP	260	75.50	202.30	118.43	25.21
WHR	260	.63	1.02	.82	.06
AGE	278	14	64	37.81	9.22
AGEONSET^2^	172	1.0	48.0	18.83	9.62
**brothers**					
BMI	90	20.99	64.82	33.67	8.08
%FAT	79	4.10	49.90	29.78	9.54
WEIGHT	89	67.05	210.11	110.27	27.48
HEIGHT	88	164.60	197.60	180.48	6.59
WAIST	88	73.10	167.00	109.44	16.65
HIP	88	80.90	175.40	112.97	16.43
WHR	88	.80	1.35	.97	.08
AGE	90	15	60	38.67	9.33
AGEONSET^2^	46	.5	45.0	22.76	10.82

1 Some obese parents (BMI>40 kg/m^2^) were selected as cases in Stage 1. All cases were independent and from different families.

2 AGEONSET refers to the age of onset for the obesity diagnosis.

**Table 3:** Top association results (P<5×10^−8^) for GWAS on obesity.

SNP	Chr	Position	Closest Gene	SNP-gene distance	MAF (case)	MAF (control)	P-value	Odds ratio	P-value (case+control+family)
rs3751812	16	52375961	*FTO*	0	0.4836	0.3632	2.01×10^−8^	1.642	4.2×10^−12^
rs8050136	16	52373776	*FTO*	0	0.4846	0.3657	3.01×10^−8^	1.631	4.7×10^−12^
rs9941349	16	52382989	*FTO*	0	0.499	0.3826	6.53×10^−8^	1.607	6.1×10^−11^
rs9930333	16	52357478	*FTO*	0	0.5144	0.3983	7.90×10^−8^	1.6	2.0×10^−11^
rs10852521	16	52362466	*FTO*	0	0.4088	0.5166	6.34×10^−7^	0.647	1.4×10^−11^
rs10891096	11	109767953	*FDX1*	38 kb	0.0904	0.0383	1.03×10^−6^	2.494	0.0045
rs7190492	16	52386253	*FTO*	0	0.3064	0.4067	1.46×10^−6^	0.644	7.8×10^−9^
rs8044769	16	52396636	*FTO*	0	0.4117	0.5139	2.36×10^−6^	0.662	4.9×10^−10^
rs9813516	3	60268044	*FHIT*	0	0.3004	0.2127	4.34×10^−6^	1.59	N/A
rs7697609	4	40651331	*APBB2*	0	0.2587	0.1766	4.71×10^−6^	1.627	0.11
rs6857327	4	40649029	*APBB2*	0	0.2658	0.1848	7.76×10^−6^	1.597	0.14
rs3912607	3	163981452	*OTOL1*	1.3 Mb	0.3721	0.4677	8.34×10^−6^	0.675	N/A
rs6921953	6	16127094	*MYLIP*	110 kb	0.0212	0.0594	9.13×10^−6^	0.344	0.00054
rs247916	5	87566009	*TMEM161B*	0	0.1965	0.2787	9.15×10^−6^	0.633	0.0022

**Table 4:** Most significantly associated SNPs in the combined case/control and family cohort.

SNP	Chr	Loc	Closest Gene	P(MQLS)
rs17817449	16	52370868	*FTO*	2.35×10^−12^
rs3751812	16	52375961	*FTO*	4.22×10^−12^
rs9935401	16	52374339	*FTO*	4.36×10^−12^
rs8050136	16	52373776	*FTO*	4.71×10^−12^
rs1121980	16	52366748	*FTO*	9.50×10^−12^
rs10852521	16	52362466	*FTO*	1.38×10^−11^
rs1861866	16	52361841	*FTO*	1.41×10^−11^
rs9937053	16	52357008	*FTO*	1.56×10^−11^
rs9930333	16	52357478	*FTO*	2.02×10^−11^
rs9931494	16	52384680	*FTO*	4.24×10^−11^
rs9941349	16	52382989	*FTO*	6.08×10^−11^
rs8044769	16	52396636	*FTO*	4.86×10^−10^
rs7206790	16	52355409	*FTO*	6.08×10^−10^
rs1477196	16	52365759	*FTO*	9.33×10^−9^
rs7190492	16	52386253	*FTO*	9.79×10^−9^
rs3751813	16	52376209	*FTO*	1.63×10^−8^
rs16867321	2	181070624	*UBE2E3*	1.63×10^−6^
rs2887180	2	181157550	*UBE2E3*	2.44×10^−6^
rs4784323	16	52355066	*FTO*	3.88×10^−6^
rs925642	4	187915860	*FAT1/MTNR1A*	7.37×10^−6^

The associated tests were performed by MQLS.

**Table 5:** List of significant associations (P<1×10^−6^) in quantitative trait analyses.

phenotype	Chr	SNP	Pos	Gene	SNP-Gene distance	P (adjusting for obesity)
BMI	8	rs17126232	18021930	*ASAH1*	35143	3.62×10^−7^
BMI	8	rs17126237	18025369	*ASAH1*	38582	5.57×10^−7^
HIP	7	rs10953454	104291049	*LHFPL3*	0	7.20×10^−7^
HIP	7	rs10216243	104300860	*LHFPL3*	0	8.07×10^−7^
HIP	8	rs17126232	18021930	*ASAH1*	35143	1.50×10^−7^
HIP	8	rs17126237	18025369	*ASAH1*	38582	3.45×10^−7^
HIP	16	rs9923451	77509940	*WWOX*	0	7.95×10^−7^
HIP	22	rs5762430	26708472	*PITPNB*	63217	7.32×10^−7^
Waist	21	rs11088859	21611215	*NCAM2*	0	3.75×10^−8^
Weight	8	rs17126232	18021930	*ASAH1*	35143	8.28×10^−8^
Weight	8	rs17126237	18025369	*ASAH1*	38582	8.74×10^−8^
Weight	13	rs17081231	65865623	*PCDH9*	0	7.18×10^−7^
WHR	2	rs7581710	120911651	*INHBB*	85798	2.12×10^−7^
WHR	6	rs2807278	131851613	*ARG1*	84445	3.18×10^−7^
WHR	14	rs11624704	77855830	*NRXN3*	84016	2.67×10^−9^

### Phenotype measures

Anthropomorphic phenotypes were directly measured in field settings. Percent fat was estimated using a bioelectric impedance (BIA) measure. The complete list of measures examined in this study is described in **Table 1** and **Figure S1**. Body mass index was calculated from measured height and weight by the standard formula, Weight (kg) divided by Height (m^2^). Measurements were taken of subjects dressed in light clothing. Height was measured from a standing position using a stadiometer. Weight was measured by a scale with a maximum weight of 600 pounds (270 kg) (Tanita TBF310 Pro Body Composition Analyzer, Tanita, Arlington Heights, IL). Body composition was estimated by bioelectric impedance using the same Tanita scale. Waist circumference was measured while standing at the height of the iliac crest. Hip circumference was taken while standing at the maximum extension of the buttocks. Waist to hip ratio (WHR) was calculated by measured waist circumference divided by measured hip circumference. Age of Obesity Onset was the age at which the subject reported having first become overweight.

### Genotyping

DNA was extracted from whole blood or lymphoblastoid cell lines using a high salt method. All cases and control subjects were genotyped on the Illumina HumanHap550 SNP arrays (Illumina, San Diego, CA) with ∼550,000 SNP markers, at the Center for Applied Genomics, Children's Hospital of Philadelphia. Standard data normalization procedures and canonical genotype clustering files were used to process the genotyping signals and generate genotype calls. In addition, the combined sample of cases, controls and family members (N = 2,256, **Table 2**) were genotyped for the top 500 SNPs from the GWAS using the Illumina ISelect platform. All cases, family members, and controls were non-Hispanic Caucasians, and we further utilized multi-dimensional scaling to confirm the ethnicity status of cases and control subjects. A subset of the whole-genome genotype data were previously described in a CNV study on obesity [23].

### Association analysis

The PLINK software version 1.07 was used to conduct association tests between SNP genotypes and specific phenotypes of interest. For traits that are approximately normally distributed, we utilized standard linear regression for assessing association but including age, sex and disease status as covariates. We attempted to exclude samples with genotyping rate less than 95% but none of the samples met this criterion. SNPs were excluded in analysis if the minor allele frequency was less than 1% (23298 SNPs were excluded), or if the Hardy-Weinberg Equilibrium P-value was less than 1×10^−6^ in control subjects (1366 SNPs were excluded), or if the genotype missing rate is higher than 5% (8190 SNPs were excluded). The study participants are of European ancestry as evaluated in previous studies [24]; given whole-genome data, we also performed multi-dimensional scaling analysis on SNPs not in LD (r^2^<0.2) with each other and confirmed that all cases and control subjects were of genetically inferred European ancestry (**Figure S2**). The QQ plot for the obesity GWAS is given in **Figure S3**, and the genomic control inflation factor was 1.05.

The combined dataset of cases, family members and controls was next analyzed using MQLS. MQLS utilizes a quasi-likelihood score test approach developed by Thornton and McPeek [25] that treats the data as a case-control analysis consisting of related and unrelated individuals. This combined approach has substantially more power than separate analyses using either case-control or family based methods. However, we acknowledge that since the candidate SNP genotyping study is not independent of the GWAS, the P-value distributions will be biased and therefore our study cannot be regarded as a standard “2-stage” analysis. The MQLS (b) statistic incorporates parental data in the estimation of case genotypes. We restricted these analyses to obesity status, since the method currently is adapted only for dichotomous phenotypes.

## Supporting Information

Figure S1 The distribution of phenotype measures utilized in the current study. The age of onset information is available for cases only. BMI, weight, BIA and waist have bi-modal distribution, so we explored testing on cases only.
(PDF)

Figure S2 Multi-dimensional scaling (MDS) of the SNP genotyping data for samples with whole-genome genotypes, with (left panel) or without (right panel) 30 Asian, 30 African American and 30 Caucasians to seed the graph. A total of 70,593 SNPs not in LD (r^2^<0.2) and not in sex chromosomes were used in the MDS analysis. All GWAS samples were of genetically inferred European ancestry.
(PDF)

Figure S3 The quantile-quantile (QQ) plot of the association results for the GWAS on obesity. The genomic control inflation factor was 1.05.
(PDF)

Table S1 Examination of BMI-associated genes in our data set for association with quantitative traits in cases and controls, adjusting for obesity status.
(PDF)

## References

[1] Haslam DW, James WP (2005). Obesity.. Lancet.

[2] Malis C, Rasmussen EL, Poulsen P, Petersen I, Christensen K (2005). Total and regional fat distribution is strongly influenced by genetic factors in young and elderly twins.. Obes Res.

[3] Stunkard AJ, Foch TT, Hrubec Z (1986). A twin study of human obesity.. Jama.

[4] Farooqi IS (2006). Genetic aspects of severe childhood obesity.. Pediatr Endocrinol Rev.

[5] Frayling TM, Timpson NJ, Weedon MN, Zeggini E, Freathy RM (2007). A common variant in the FTO gene is associated with body mass index and predisposes to childhood and adult obesity.. Science.

[6] Loos RJ, Lindgren CM, Li S, Wheeler E, Zhao JH (2008). Common variants near MC4R are associated with fat mass, weight and risk of obesity.. Nat Genet.

[7] Scherag A, Dina C, Hinney A, Vatin V, Scherag S (2010). Two New Loci for Body-Weight Regulation Identified in a Joint Analysis of Genome-Wide Association Studies for Early-Onset Extreme Obesity in French and German Study Groups.. PLoS Genet.

[8] Willer CJ, Speliotes EK, Loos RJ, Li S, Lindgren CM (2009). Six new loci associated with body mass index highlight a neuronal influence on body weight regulation.. Nat Genet.

[9] Lindgren CM, Heid IM, Randall JC, Lamina C, Steinthorsdottir V (2009). Genome-wide association scan meta-analysis identifies three Loci influencing adiposity and fat distribution.. PLoS Genet.

[10] Thorleifsson G, Walters GB, Gudbjartsson DF, Steinthorsdottir V, Sulem P (2009). Genome-wide association yields new sequence variants at seven loci that associate with measures of obesity.. Nat Genet.

[11] Heid IM, Jackson AU, Randall JC, Winkler TW, Qi L (2010). Meta-analysis identifies 13 new loci associated with waist-hip ratio and reveals sexual dimorphism in the genetic basis of fat distribution.. Nat Genet.

[12] Heard-Costa NL, Zillikens MC, Monda KL, Johansson A, Harris TB (2009). NRXN3 is a novel locus for waist circumference: a genome-wide association study from the CHARGE Consortium.. PLoS Genet.

[13] Speliotes EK, Willer CJ, Berndt SI, Monda KL, Thorleifsson G (2010). Association analyses of 249,796 individuals reveal 18 new loci associated with body mass index.. Nat Genet.

[14] Cotsapas C, Speliotes EK, Hatoum IJ, Greenawalt DM, Dobrin R (2009). Common body mass index-associated variants confer risk of extreme obesity.. Hum Mol Genet.

[15] Meyre D, Delplanque J, Chevre JC, Lecoeur C, Lobbens S (2009). Genome-wide association study for early-onset and morbid adult obesity identifies three new risk loci in European populations.. Nat Genet.

[16] Hinney A, Nguyen TT, Scherag A, Friedel S, Bronner G (2007). Genome wide association (GWA) study for early onset extreme obesity supports the role of fat mass and obesity associated gene (FTO) variants.. PLoS ONE.

[17] Walters RG, Jacquemont S, Valsesia A, de Smith AJ, Martinet D (2010). A new highly penetrant form of obesity due to deletions on chromosome 16p11.2.. Nature.

[18] Willer CJ, Sanna S, Jackson AU, Scuteri A, Bonnycastle LL (2008). Newly identified loci that influence lipid concentrations and risk of coronary artery disease.. Nat Genet.

[19] Rowen L, Young J, Birditt B, Kaur A, Madan A (2002). Analysis of the Human Neurexin Genes: Alternative Splicing and the Generation of Protein Diversity.. Genomics.

[20] Hishimoto A, Liu QR, Drgon T, Pletnikova O, Walther D (2007). Neurexin 3 polymorphisms are associated with alcohol dependence and altered expression of specific isoforms.. Hum Mol Genet.

[21] Lachman HM, Fann CSJ, Bartzis M, Evgrafov OV, Rosenthal RN (2007). Genomewide suggestive linkage of opioid dependence to chromosome 14q.. Human Molecular Genetics.

[22] Novak G, Boukhadra J, Shaikh SA, Kennedy JL, Le Foll B (2009). Association of a polymorphism in the NRXN3 gene with the degree of smoking in schizophrenia: a preliminary study.. World J Biol Psychiatry.

[23] Wang K, Li WD, Glessner JT, Grant SF, Hakonarson H (2010). Large copy number variations are enriched in cases with moderate to extreme obesity.. Diabetes.

[24] Price RA, Li WD, Zhao H (2008). FTO gene SNPs associated with extreme obesity in cases, controls and extremely discordant sister pairs.. BMC Med Genet.

[25] Thornton T, McPeek MS (2007). Case-control association testing with related individuals: a more powerful quasi-likelihood score test.. Am J Hum Genet.

