# Correction: A Genome-Wide Association Study on Obesity and Obesity-Related Traits

**DOI:** 10.1371/annotation/a34ee94e-3e6a-48bd-a19e-398a4bb88580

**Published:** 2012-02-23

**Authors:** Kai Wang, Wei-Dong Li, Clarence K. Zhang, Zuoheng Wang, Joseph T. Glessner, Struan F. A. Grant, Hongyu Zhao, Hakon Hakonarson, R. Arlen Price

There was an error in the funding statement. The correct funding statement is: "This work was supported in part by NIH grants R01DK44073, R01DK56210, and R01DK076023 to R.A.P. and a Scientist Development Grant (0630188N) from the American Heart Association to W.D.L. Genome-wide genotyping was funded in part by an Institutional Development Award to the Center for Applied Genomics (H.H.) from the Children's Hospital of Philadelphia. Follow-up genotyping after the initial GWAS was funded in part by the Center for Inherited Disease Research (CIDR). CIDR is funded through a federal contract from the U.S. National Institutes of Health to The Johns Hopkins University, contract number HHSN268200782096C. No additional external funding was received for this study. The funders had no role in study design, data collection and analysis, decision to publish, or preparation of the manuscript." 

