# Supplementary material for: A Genome-Wide Association Study on Obesity and Obesity-Related Traits
Source: PLoS One. 2011 Apr 28;6(4):e18939. doi: 10.1371/journal.pone.0018939 (PMC3084240; doi:10.1371/journal.pone.0018939)
Supplement: Figure S1 — The distribution of phenotype measures utilized in the current study. The age of onset information is available for cases only. BMI, weight, BIA and waist have bi-modal distribution, so we explored testing on cases only. (PDF) [file pone.0018939.s001.pdf]

Figure S1. The distribution of phenotype measures utilized in the current study. The age of onset information is available for cases only.

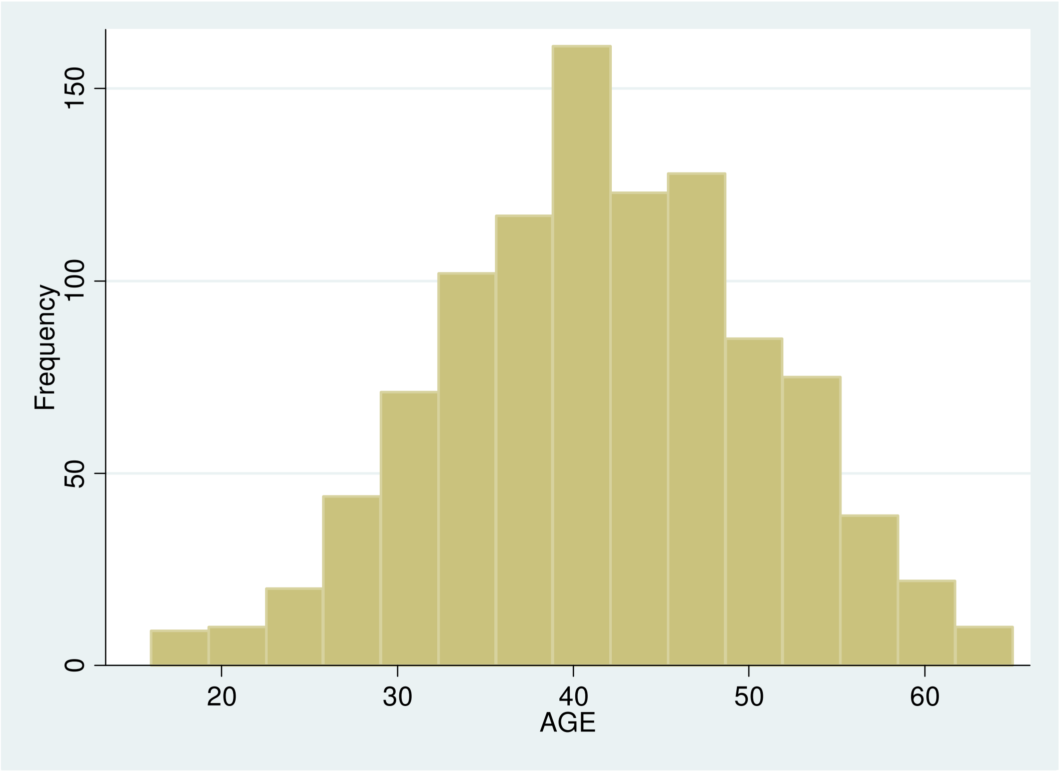

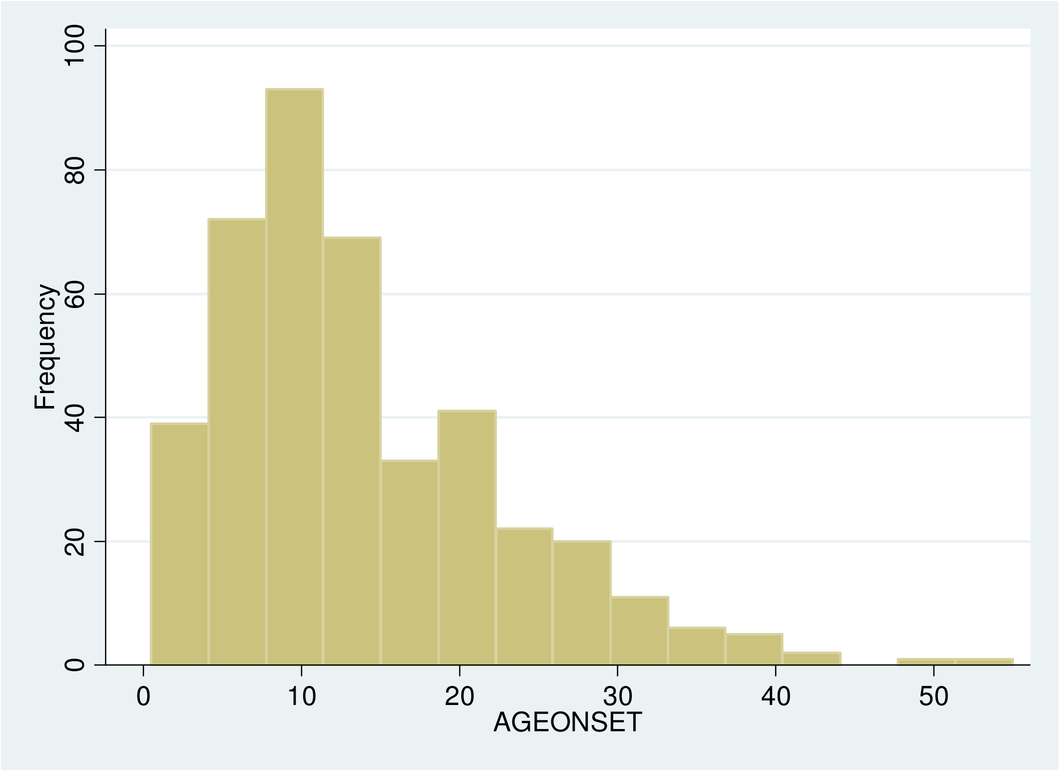

Frequency
AGEONSET
0
20
40
60
80
100
0
10
20
30
40
50

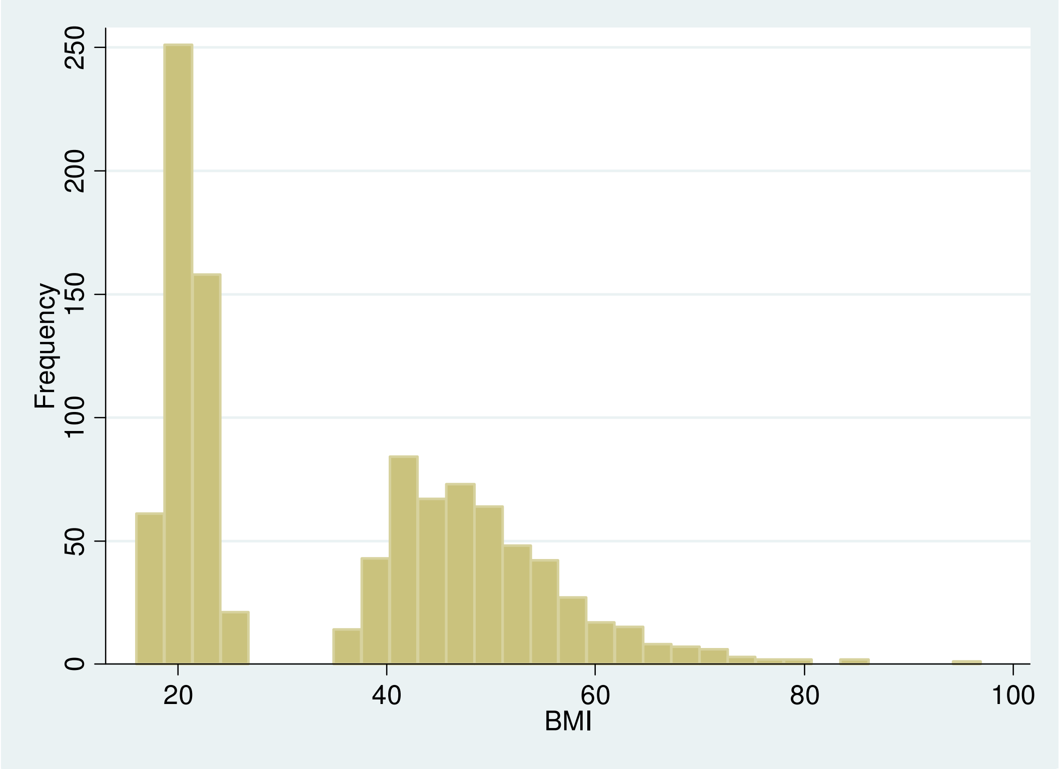

Frequency
0
50
100
150
200
250
20
40
60
80
100
BMI

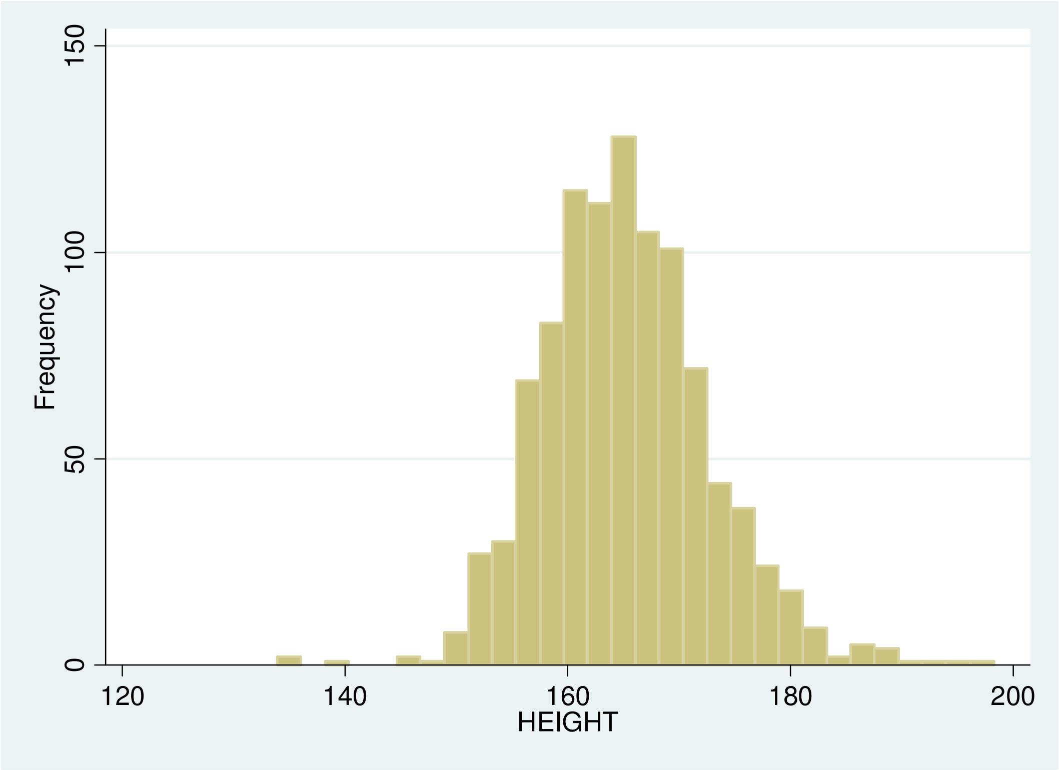

150
100
50
0
Frequency
120
140
160
180
200
HEIGHT

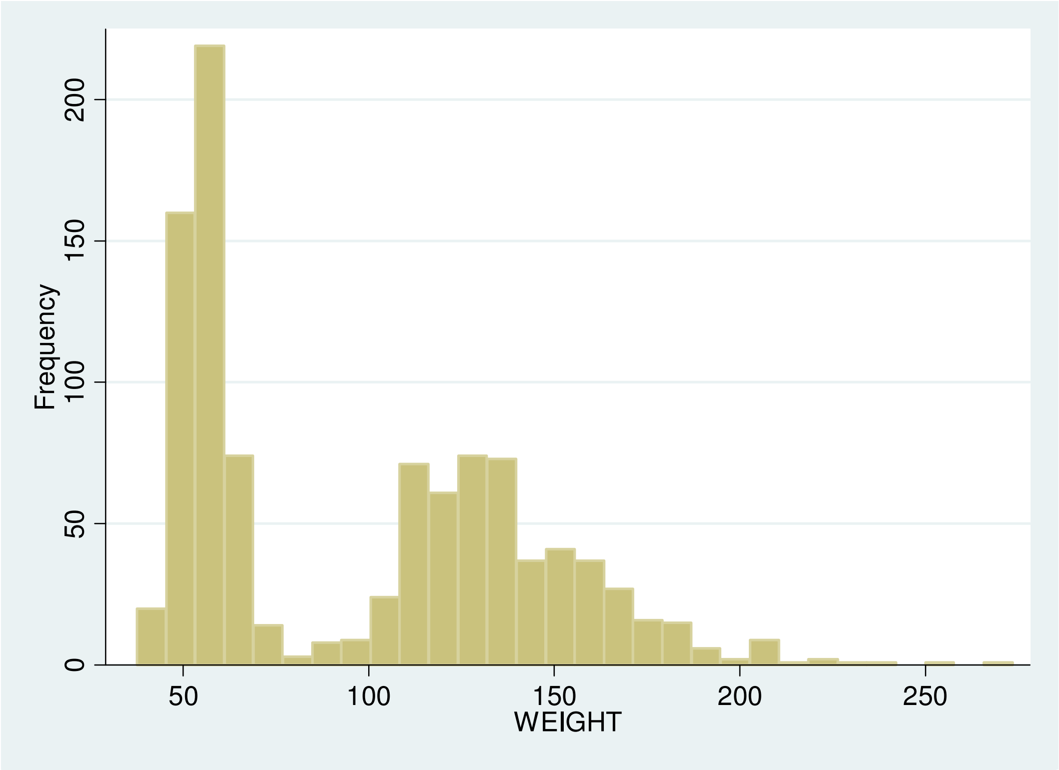

Frequency
0
50
100
150
200
50
100
150
200
250
WEIGHT

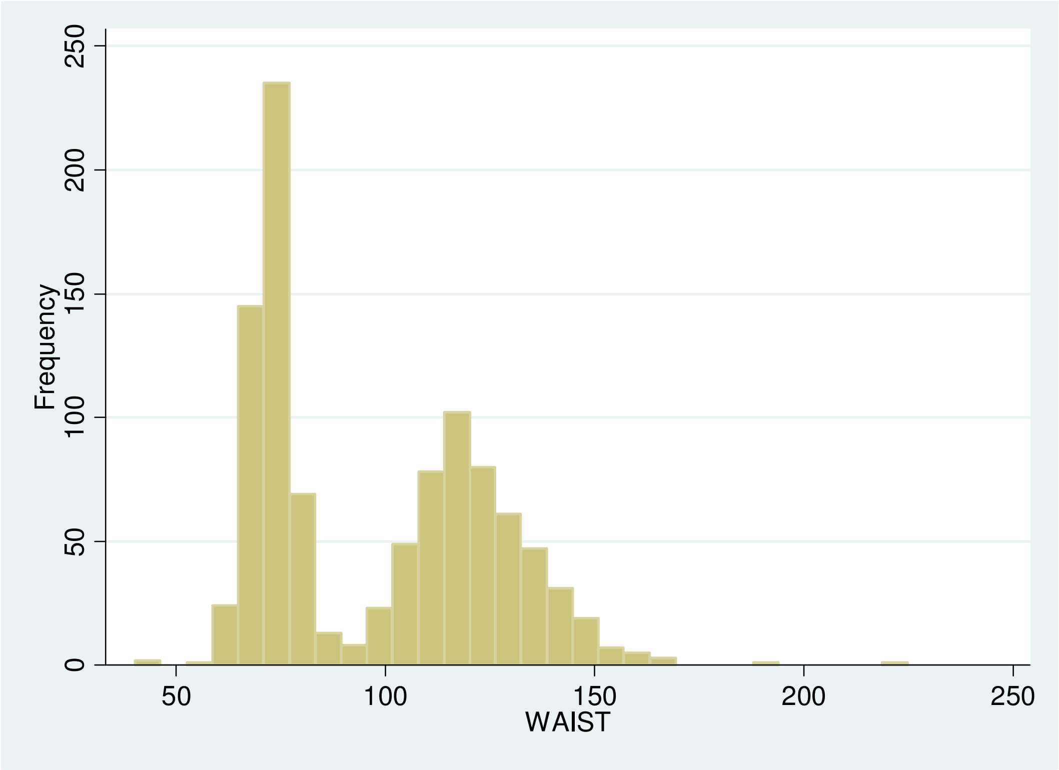

Frequency
0
50
100
150
200
250
50
100
150
200
250
WAIST

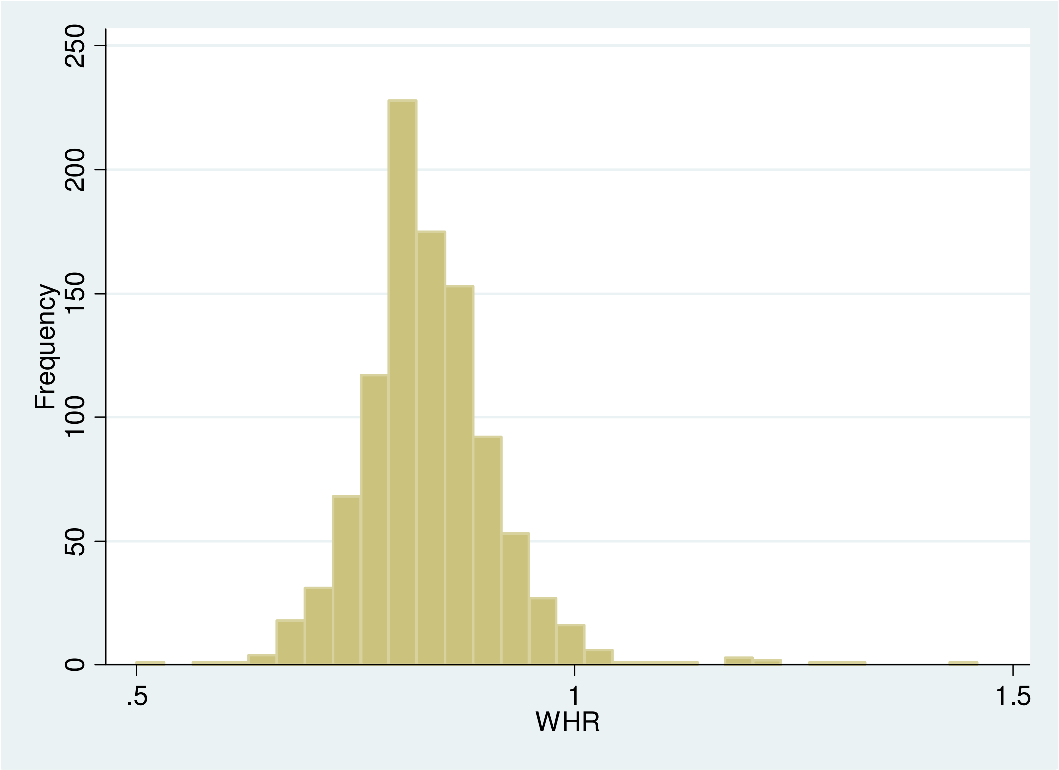

Frequency
0
50
100
150
200
250
.5
1
1.5
WHR

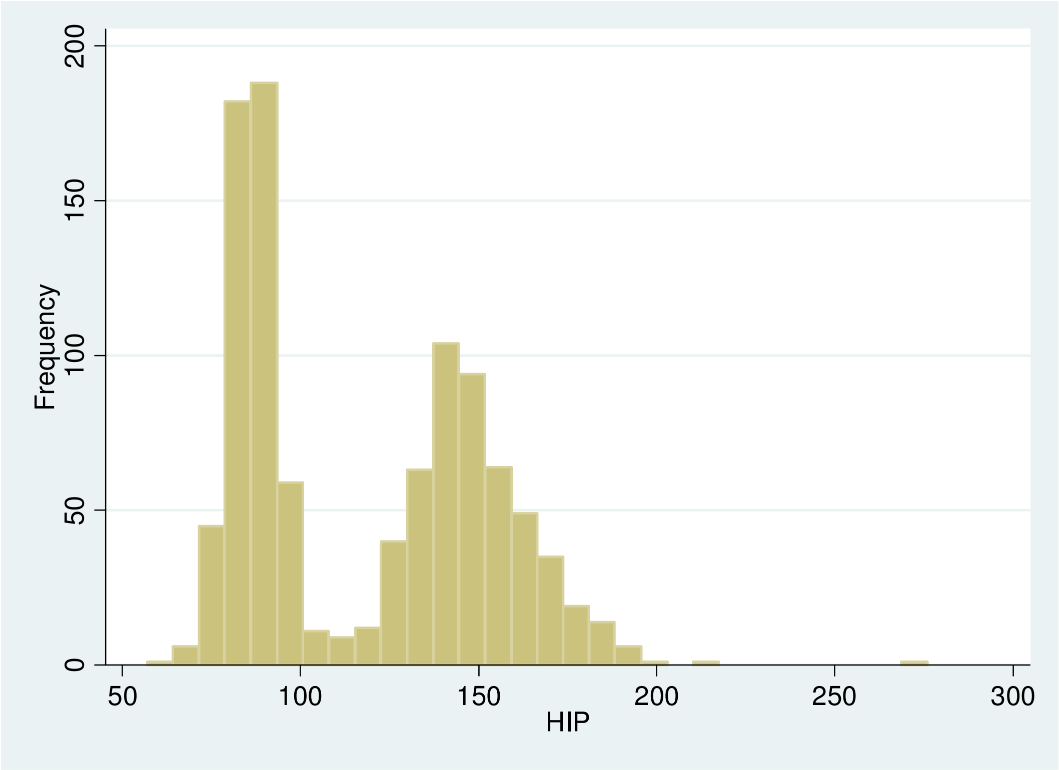

200
150
100
50
0
Frequency
50
100
150
200
250
300
HIP

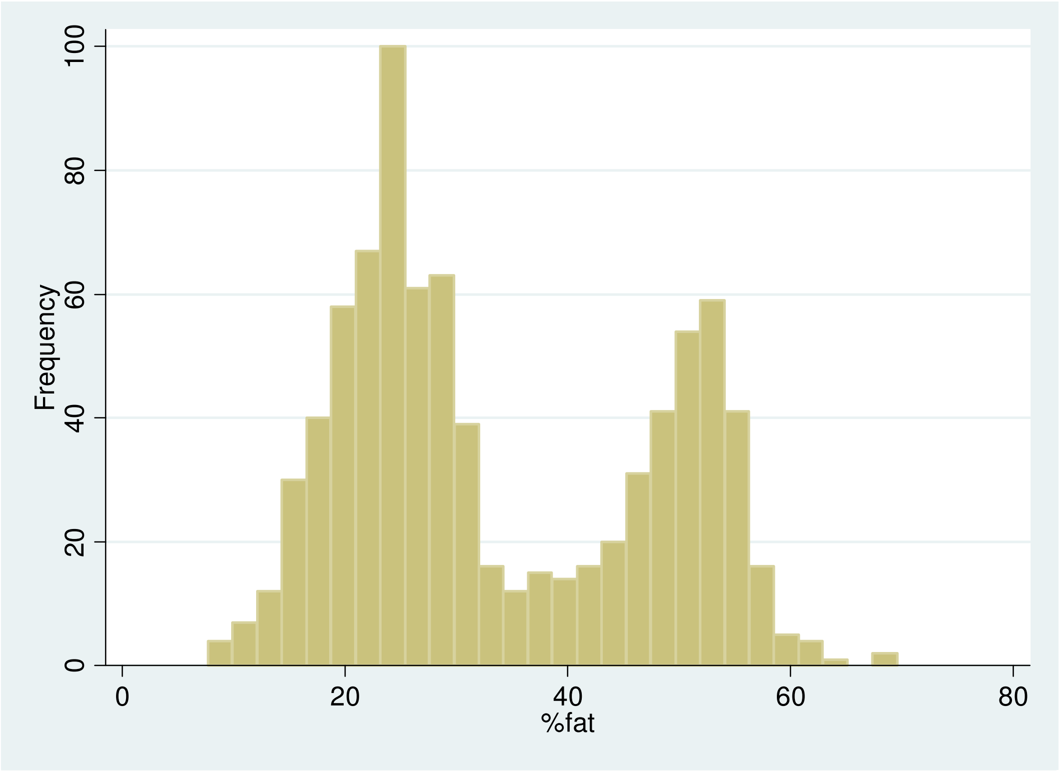

Frequency
0
20
40
60
80
100
0
20
40
60
80
%fat
