# Supplementary material for: A Genome-Wide Association Study on Obesity and Obesity-Related Traits
Source: PLoS One. 2011 Apr 28;6(4):e18939. doi: 10.1371/journal.pone.0018939 (PMC3084240; doi:10.1371/journal.pone.0018939)

Figure S2. Multi-dimensional scaling (MDS) of the SNP genotyping data for samples with whole-genome genotypes, with (left panel) or without (right panel) 30 Asian, 30 African American and 30 Caucasians to seed the graph. A total of 70593 SNPs not in LD (r^2<0.2) and not in sex chromosomes were used in the MDS analysis. All GWAS samples were of genetically inferred European ancestry.

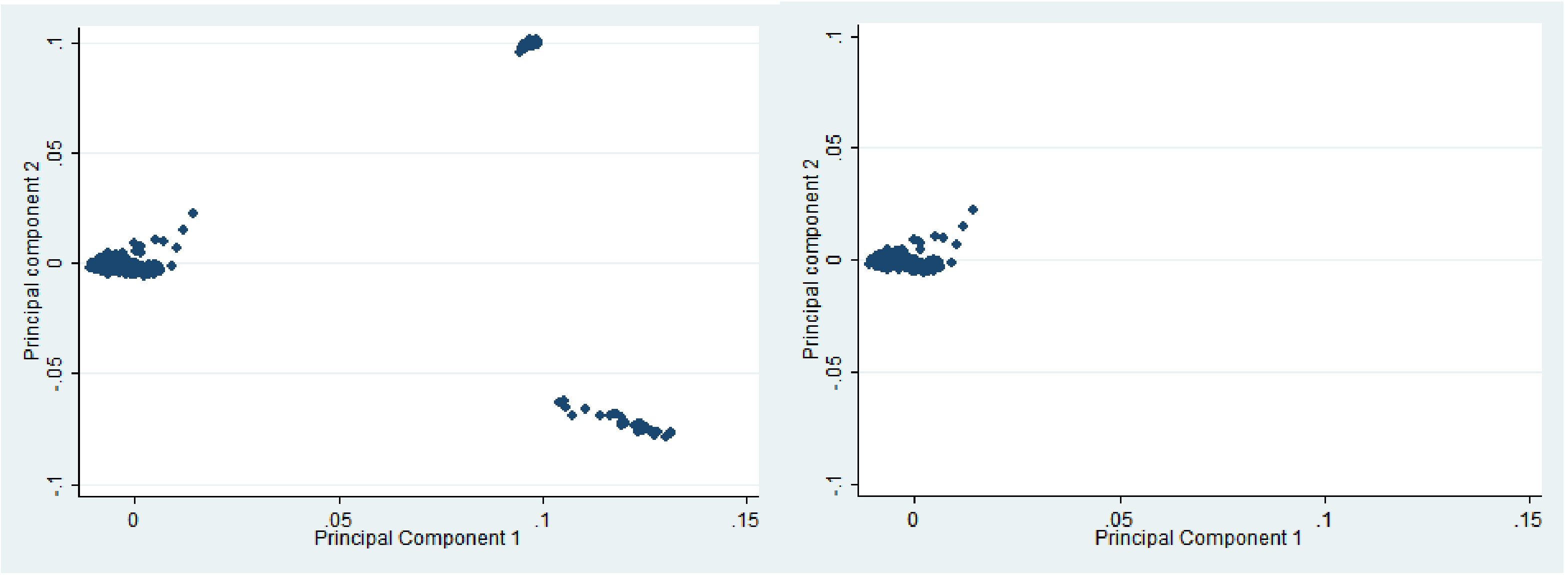

Supplement: Figure S2 — Multi-dimensional scaling (MDS) of the SNP genotyping data for samples with whole-genome genotypes, with (left panel) or without (right panel) 30 Asian, 30 African American and 30 Caucasians to seed the graph. A total of 70,593 SNPs not in LD (r2<0.2) and not in sex chromosomes were used in the MDS analysis. All GWAS samples were of genetically inferred European ancestry. (PDF) [file pone.0018939.s002.pdf]
