# Supplementary material for: A Genome-Wide Association Study on Obesity and Obesity-Related Traits
Source: PLoS One. 2011 Apr 28;6(4):e18939. doi: 10.1371/journal.pone.0018939 (PMC3084240; doi:10.1371/journal.pone.0018939)

Figure S3. The quantile-quantile (QQ) plot of the association results for the GWAS on obesity. The genomic control inflation factor was 1.05.

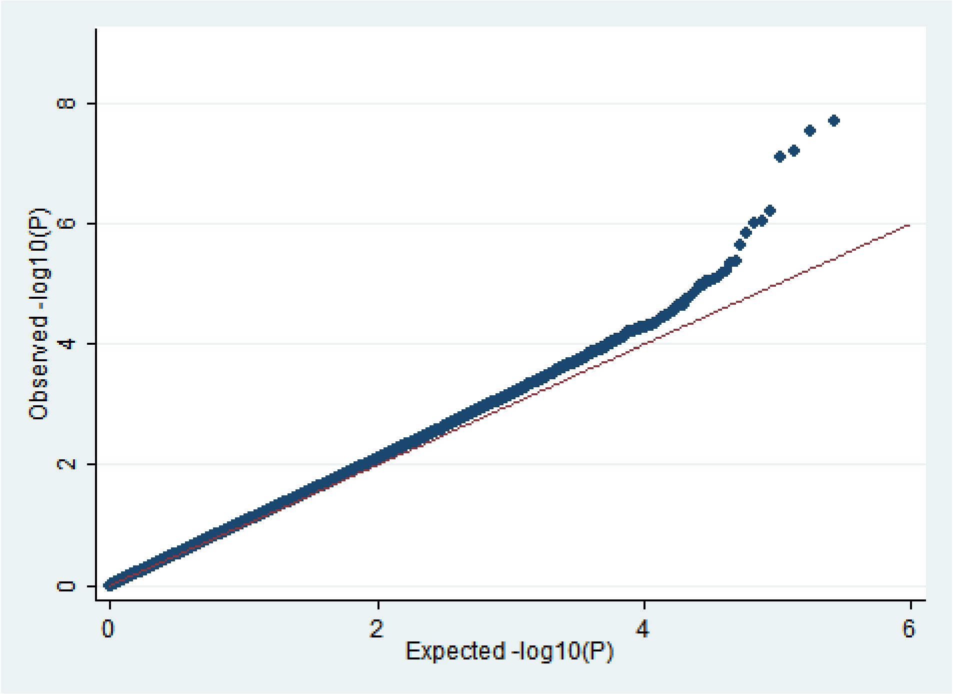

Supplement: Figure S3 — The quantile-quantile (QQ) plot of the association results for the GWAS on obesity. The genomic control inflation factor was 1.05. (PDF) [file pone.0018939.s003.pdf]
