# Supplementary material for: A Genome-Wide Association Study on Obesity and Obesity-Related Traits
Source: PLoS One. 2011 Apr 28;6(4):e18939. doi: 10.1371/journal.pone.0018939 (PMC3084240; doi:10.1371/journal.pone.0018939)
Supplement: Table S1 — Examination of BMI-associated genes in our data set for association with quantitative traits in cases and controls, adjusting for obesity status. (PDF) [file pone.0018939.s004.pdf]

Table S1. Examination of BMI-associated genes in our data set for association with quantitative traits in cases and controls, adjusting for obesity status.

Nearest gene	Other nearby genes	Chr.	SNP	Position (bp)	best proxy	r2	distance	P(obesity)	OR(obesity)	P(height)	P(weight)	P(bia)	P(waist)	P(whr)	P(hip)
FTO		16	rs1558902	52,361,075	rs8050136	0.9065	12701	3.01E-08	1.631	0.4674	0.2192	0.6905	0.2145	0.4806	0.1736
TMEM18		2	rs2867125	612,827	rs2867125	1	0	0.1223	0.8388	0.7049	0.6322	0.7437	0.6887	0.4836	0.7922
MC4R (B)		18	rs571312	55,990,749	rs571312	1	0	0.02334	1.26	0.3765	0.6043	0.7812	0.6561	0.4731	0.7019
GNPDA2		4	rs10938397	44,877,284	rs12641981	1	2644	0.7471	1.029	0.06339	0.008078	0.2353	0.9716	0.2856	0.1959
BDNF (B,M)		11	rs10767664	27,682,562	rs11030104	0.9036	41469	0.12	0.847	0.4592	0.1125	0.5552	0.1656	0.9517	0.06438
NEGR1 (C,Q)		1	rs2815752	72,585,028	rs2568958	1	47324	0.6819	0.9634	0.9705	0.2706	0.1162	0.07191	0.2176	0.4925
SH2B1 (Q,B,M)	APOB48R (Q,M), SULT1A2 (Q,M), AC138894.2 (M), ATXN2L (M), TUFM (Q)	16	rs7359397	28,793,160	rs7498665	1	2418	0.0653	1.178	0.8327	0.5653	0.0464	0.9674	0.09441	0.3487
ETV5		3	rs9816226	187,317,193	rs7647305	0.807	209	0.06872	0.8216	0.4792	0.5914	0.8901	0.4387	0.08986	0.6256
MTCH2 (Q,M)	NDUFS3 (Q), CUGBP1 (Q)	11	rs3817334	47,607,569	rs7124681	1	121046	0.2104	1.117	0.4465	0.8086	0.7352	0.8446	0.2076	0.1826
KCTD15		19	rs29941	39,001,372	rs29941	1	0	0.02433	0.8059	0.8503	0.8869	0.6712	0.4053	0.3129	0.6239
SEC16B		1	rs543874	176,156,103	rs10913469	0.9575	24039	0.3484	1.111	0.3545	0.9232	0.4456	0.5619	0.7035	0.1907
TFAP2B		6	rs987237	50,911,009	rs987237	1	0	0.1107	1.198	0.1346	0.8453	0.1353	0.2469	0.8108	0.2528
FAIM2		12	rs7138803	48,533,735	rs7138803	1	0	0.2677	1.103	0.9558	0.9481	0.07815	0.6593	0.9139	0.7134
NRXN3		14	rs10150332	79,006,717	rs10146997	1	8198	0.2991	1.114	0.41	0.6851	0.4047	0.5499	0.5124	0.3535
RBJ	ADCY3 (Q, M), POMC (Q,B)	2	rs713586	25,011,512	rs713587	1	273	0.3283	1.089	0.8767	0.5664	0.9422	0.4515	0.5074	0.5697

GPRC5B (C,Q)	IQCK (Q)	16	rs12444979	19,841,101	rs12446632	1	1789	0.01604	0.7379	0.1159	0.7166	0.2257	0.694	0.2639	0.5852
MAP2K5	LBXCOR1 (M)	15	rs2241423	65,873,892	rs2241423	1	0	0.002696	0.7303	0.5409	0.6476	0.9151	0.4375	0.8233	0.6929
QPCTL	GIPR (B,M)	19	rs2287019	50,894,012	rs2287019	1	0	0.05068	0.8019	0.5813	0.7199	0.6238	0.2846	0.1024	0.6045
TNNI3K		1	rs1514175	74,764,232	rs1514175	1	0	0.4353	1.071	0.6313	0.4371	0.4761	0.1004	0.4499	0.3289
SLC39A8 (Q,M)		4	rs13107325	103,407,732	rs13107325	1	0	0.9365	0.9868	0.5014	0.9357	0.7016	0.5628	0.04951	0.4287
FLJ35779 (M)	HMGCR (B)	5	rs2112347	75,050,998	rs2047059	0.8296	7049	0.8482	0.9816	0.29	0.9109	0.1976	0.1133	0.07325	0.8464
LRRN6C		9	rs10968576	28,404,339	rs10968576	1	0	0.7195	0.9668	0.2167	0.0734	0.2794	0.6638	0.6932	0.1487
TMEM160 (Q)	ZC3H4 (Q)	19	rs3810291	52,260,843	rs2303108	0.8454	20892	0.2529	0.897	0.2096	0.1116	0.6776	0.5941	0.4263	0.7249
FANCL		2	rs887912	59,156,381	rs759250	1	26276	0.000551	1.387	0.6009	0.5726	0.958	0.5221	0.09635	0.4439
CADM2		3	rs13078807	85,966,840	rs13078807	1	0	0.07795	1.217	0.3018	0.4706	0.6164	0.7078	0.1049	0.2401
PRKD1		14	rs11847697	29,584,863	rs225890	0.3287	46570	0.5546	1.067	0.01182	0.7319	0.0761	0.6185	0.1857	0.7199
LRP1B		2	rs2890652	142,676,401	rs17832932	0.6133	17136	0.4286	1.117	0.7638	0.2578	0.4196	0.05611	0.8736	0.07389
PTBP2		1	rs1555543	96,717,385	rs11165643	1	20700	0.6363	0.9594	0.749	0.4581	0.5193	0.8092	0.4464	0.5425
MTIF3	GTF3A (Q)	13	rs4771122	26,918,180	rs1475221	0.8473	17386	0.3547	0.9051	0.6503	0.2815	0.9787	0.3287	0.7488	0.2022
RPL27A	TUB (B)	11	rs4929949	8,561,169	rs10840065	0.9496	77950	0.8248	1.019	0.2155	0.5686	0.7093	0.9583	0.1758	0.515
NUDT3	HMGA1 (B)	6	rs206936	34,410,847	rs464553	1	12210	0.9535	1.006	0.734	0.8497	0.8531	0.6658	0.8315	0.3916
ZNF608		5	rs4836133	124,360,002	NONE	NA	NA								
